# Gut Microbiome Changes in Preclinical Alzheimer’s Disease

**DOI:** 10.3390/nu18152469

**Published:** 2026-07-29

**Authors:** D. M. Sithara Dissanayaka, Stephanie R. Rainey-Smith, Hamid R. Sohrabi, Thilini N. Jayasinghe, Vincent Ho, Vijay Jayasena, Kevin Taddei, Colin L. Masters, Ralph N. Martins, W. M. A. D. Binosha Fernando

**Affiliations:** 1 Centre of Excellence for Alzheimer’s Disease Research & Care, School of Medical and Health Sciences, Edith Cowan University, Joondalup, WA 6027, Australia; sitharad@our.ecu.edu.au (D.M.S.D.);; 2 Alzheimer’s Research Australia, Ralph and Patricia Sarich Neuroscience Research Institute, Nedlands, WA 6009, Australia; 3 Centre for Healthy Ageing, Murdoch University, Murdoch, WA 6150, Australia; 4 Department of Biomedical Sciences, Faculty of Medicine, Health and Human Sciences, Macquarie University, Sydney, NSW 2109, Australia; 5 Sydney Dental School, Faculty of Medicine and Health, University of Sydney, Camperdown, NSW 2050, Australia; 6 Oral Health Services, Western Sydney Local Health District, Westmead, NSW 2151, Australia; 7 School of Medicine, Western Sydney University, Campbelltown Hospital, Campbelltown, NSW 2560, Australia; 8 School of Science and Health, Western Sydney University, M15, Room G54, Locked Bag 1797, Penrith, NSW 2751, Australia; 9 Medical School, University of Western Australia, Perth, WA 6009, Australia; 10 The Florey Institute of Neuroscience and Mental Health, University of Melbourne, Melbourne, VIC 3052, Australia

**Keywords:** Alzheimer’s disease, gut microbiome, short-chain fatty acids, dietary patterns, preclinical, gut–brain axis, neuroinflammation

## Abstract

Alzheimer’s disease (AD) is a progressive neurodegenerative disorder that develops many years before clinical symptoms appear. The biological changes involved in the earliest stages remain poorly understood, particularly during the preclinical stage. Our previous work has identified gradual gut microbial and metabolic changes during this stage, suggesting these may represent early biological shifts that precede disease progression. Recent studies suggest that the gut microbiome may contribute to early AD processes through its effects on immune regulation, metabolism, and gut–brain communication. Changes in gut microbial composition, including reduced levels of short-chain fatty acid (SCFA)-producing bacteria, such as *Faecalibacterium*, *Roseburia*, and *Eubacterium*, have been reported in individuals with AD and mild cognitive impairment. These microbial alterations have also been linked to disrupted metabolic activity, impaired gut barrier function, and increased neuroinflammatory responses. Diet is an important factor influencing gut microbial composition and metabolic activity. Mediterranean, DASH, and prudent dietary patterns are generally associated with beneficial microbial profiles and increased SCFA production, whereas Western dietary patterns are linked to lower microbial diversity and increased pro-inflammatory taxa. This review summarises the current evidence linking gut microbiota, SCFAs, microbial metabolism, and dietary patterns with early AD pathology, while highlighting important gaps in the existing literature.

## 1. Introduction

Alzheimer’s disease (AD) is a neurodegenerative disorder with a long preclinical stage, defined as the period during which neuropathological changes are present before the onset of clinically detectable cognitive impairment [1]. During this stage, processes such as amyloid accumulation, neuroinflammation, and metabolic alterations develop while individuals remain cognitively unimpaired [2,3]. Therefore, understanding the changes that occur during the preclinical stage is critical for elucidating mechanisms relevant to early prevention.

Emerging evidence indicates that early AD pathology is accompanied by systemic changes, including alterations in the gut microbiota. Accordingly, differences in gut microbial composition have been reported in individuals with AD and mild cognitive impairment, as well as in populations considered at increased risk of disease. The pattern of these differences is not yet consistent across studies, and most available evidence is cross-sectional, meaning it remains unclear whether microbial changes precede AD pathology or occur as a secondary consequence of early disease processes [4,5]. These compositional changes are characterised by a reduced abundance of short-chain fatty acid (SCFA) producing taxa and accompanying changes in microbial metabolic activity. Such alterations suggest the disruption of gut-derived metabolic functions that are important for maintaining intestinal and immune homeostasis [6,7].

SCFAs, including acetate, propionate, and butyrate, play key roles in maintaining intestinal barrier integrity, regulating immune responses, and supporting gut–brain communication. Consistent with this, alterations in SCFA related pathways have been linked to neuroinflammatory responses and amyloid-related mechanisms associated with AD pathology [8,9,10]. However, much of the existing evidence is derived from symptomatic populations, and studies characterising microbial and metabolic alterations during the preclinical stage of AD, particularly in relation to underlying pathology, remain limited, with only a small number of studies including cognitively unimpaired biomarker-confirmed cohorts.

Diet plays a key role in gut microbial composition and metabolic output. By modulating the substrate availability for microbial fermentation, diet influences microbial composition and pathways involved in carbohydrate metabolism, lipid metabolism, and inflammatory signalling within the gut ecosystem [11,12]. These diet-driven microbial changes, in turn, affect gut–brain communication processes that are relevant to neuroinflammatory and metabolic regulation [13]. In parallel, increasing evidence links long term dietary patterns with cognitive impairment and AD risk, highlighting diet as an important modifiable factor in neurodegenerative disease [14]. Together, these observations suggest that dietary exposures may influence AD-related biological mechanisms from the earliest stages of disease development. However, despite diet microbiome interactions, their relevance to microbial functional pathways during the preclinical stage of AD remains poorly characterised [15,16].

The present work addresses these gaps by examining gut microbial composition, faecal SCFA profiles, microbial metabolic pathway alterations, and dietary patterns in individuals at the preclinical stage of AD. Notably, this work provides a systematic evaluation of these gut-related measures in relation to early AD pathology within a cognitively unimpaired cohort, enabling assessment of the association between gut microbial composition and early AD pathology.

## 2. Alzheimer’s Disease

AD is the most common form of dementia and is characterised by progressive cognitive decline, neuropsychiatric changes, and functional impairment that ultimately result in loss of independence and death [17]. Dementia affects more than 55 million people globally, with prevalence expected to increase substantially as populations age [18]. AD accounts for approximately 60–80% of all dementia cases and is a major cause of morbidity and mortality/morbidity and mortality worldwide, with the majority of the projected increase in disease burden occurring in low- and middle-income countries as populations age [19].

**Figure 1:**
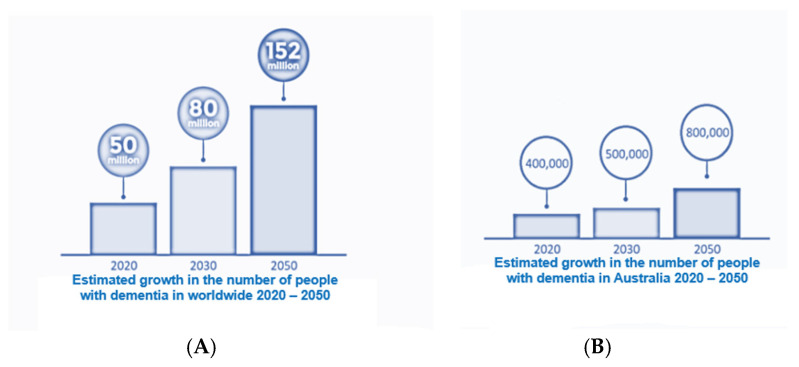
Expected growth in the number of people living with dementia globally and in Australia. (**A**) Estimated global increase in the number of people living with dementia from 2020 to 2050. (**B**) Estimated increase in the number of people living with dementia in Australia over the same period.

The societal and economic burden of dementia is substantial. Globally, the cost of dementia related care exceeds USD 1 trillion annually [20]. In Australia, more than 400,000 people are currently living with dementia, and annual health system expenditure related to dementia care exceeds AUD 3.7 billion, with a large proportion attributable to residential aged care (Figure 1) [18,21,22]. As cognitive and functional impairment progresses, individuals increasingly rely on informal caregivers and formal care systems, contributing to reduced workforce participation and escalating societal costs [23].

Given this substantial and growing burden, effective disease modifying therapies for AD are urgently needed. However, despite extensive research efforts, available disease-modifying treatments have demonstrated limited clinical benefit. A major reason for this is that diagnosis and treatment typically occur only after substantial neurodegeneration has already developed [24]. Consequently, therapeutic interventions are frequently initiated at a stage when disease pathology is already well established, limiting their potential effectiveness [25,26,27]. This highlights the need to better understand the biological changes that occur prior to the onset of clinical symptoms [27,28].

### Clinical Progression of Alzheimer’s Disease

AD develops over many years before clinical symptoms become apparent. During this extended preclinical phase, neuropathological changes accumulate while individuals remain cognitively unimpaired despite the presence of underlying brain pathology [29]. This stage is characterised by early disease processes include amyloid-beta (Aβ) accumulation, tau pathology, synaptic dysfunction, and progressive neuronal loss [30,31]. Preclinical AD is, therefore, defined by biomarker evidence of pathology, commonly identified using positron emission tomography (PET) imaging or cerebrospinal fluid (CSF) measures of Aβ [32,33]. Emerging evidence suggests that alterations in gut microbial composition may also be detectable during this preclinical stage.

As pathological burden increases, some individuals transition to mild cognitive impairment (MCI), a clinical stage characterised by cognitive decline exceeding age-related changes while functional independence is largely maintained [24]. Disease progression beyond MCI is heterogeneous, with only a subset of individuals progressing to AD dementia. Individuals who progress to AD dementia experience decline across multiple cognitive domains accompanied by functional impairment and the emergence of neuropsychiatric symptoms [24,31,34,35]. Increasing evidence indicates that alterations in gut microbial composition and function are also observed in individuals with MCI and AD [34]. These gut microbial changes may occur across disease stages and are thought to interact with inflammatory, metabolic, and immune pathways relevant to AD [36,37].

## 3. Pathophysiology of Alzheimer’s Disease

AD is characterised by a progressive accumulation of neuropathological changes in the brain over many years. Hallmark pathological features include the deposition of extracellular Aβ plaques and the formation of intracellular neurofibrillary tangles composed of hyperphosphorylated tau protein [38]. These abnormalities preferentially affect brain regions involved in memory and higher cognitive processing, including the hippocampus and associative cortices, and are closely associated with synaptic dysfunction, neuronal loss, and progressive cognitive decline [30].

Several hypotheses have been proposed to describe how these pathological changes develop and interact during AD progression. The amyloid cascade hypothesis proposes that abnormal accumulation of Aβ characterises an early pathological event that initiates downstream processes, including tau pathology, neuroinflammation, synaptic dysfunction, and neurodegeneration [39,40]. At this stage, individuals may already demonstrate cerebral amyloid accumulation while remaining cognitively unimpaired. In contrast, the tau hypothesis emphasises tau pathology as a more direct driver of neuronal dysfunction and cognitive decline, supported by evidence that tau burden correlates more closely with disease severity and clinical symptoms than amyloid deposition [41]. These hypotheses are not mutually exclusive; rather, they describe interacting pathological processes in which amyloid accumulation, tau pathology, and neuroinflammation influence one another during disease progression [42,43].

Alongside amyloid and tau pathology, neuroinflammation is another key pathological feature of AD. Chronic activation of innate immune responses, particularly involving microglia and astrocytes, is consistently observed in brain regions affected by AD [44]. This sustained glial activation promotes the release of pro-inflammatory cytokines, chemokines, and reactive oxygen species, which contribute to ongoing synaptic dysfunction and neuronal injury. Immune dysregulation in AD is further supported by genetic studies identifying multiple risk genes associated with immune and microglial function [45,46].

Metabolic dysfunction also develops alongside inflammatory- and protein-related pathology and further contributes to neuronal vulnerability in AD [47]. Neurons in AD exhibit impaired mitochondrial function, reduced energy metabolism, and increased production of reactive oxygen species, leading to progressive cellular stress and damage [48]. This oxidative environment further enhances both amyloid and tau pathology, thereby promoting neurodegenerative processes [49]. In addition, disturbances in calcium homeostasis and axonal transport also impair neuronal signalling and viability, contributing to progressive synaptic deterioration. Together, these metabolic disturbances accelerate neuronal dysfunction and contribute to the progressive loss of cognitive function observed in AD [50].

Although AD pathology is in the brain, increasing evidence indicates that systemic and peripheral processes also contribute to disease development and progression [51]. Vascular dysfunction, metabolic abnormalities, and altered peripheral immune signalling have all been associated with increased AD risk and accelerated neurodegeneration [8]. These systemic disturbances can influence cerebral homeostasis by modifying inflammatory and metabolic pathways that affect neuronal function. Within this broader context, peripheral systems such as the gut–brain axis have gained attention as potential modulators of neuroinflammatory and metabolic signalling relevant to AD [52,53].

While the mechanisms linking gut-derived signals to brain pathology are not yet fully defined, these observations highlight the multifactorial nature of AD and emphasise that disease progression is driven by interactions between central and peripheral biological processes [36,54,55].

### 3.1. Amyloid-Beta Peptides

Aβ peptides are produced by sequential cleavage of the amyloid precursor protein (APP) by β-secretase and γ-secretase enzymes [56]. Among the Aβ species produced, the Aβ42 isoform is particularly important due to its increased hydrophobicity and strong tendency to misfold and self-associate [57,58]. This preferential accumulation of Aβ42 promotes the formation of soluble oligomeric assemblies, which are considered the most neurotoxic Aβ species [59,60]. They result in the development of fibrillar aggregates and extracellular amyloid plaques within the brain parenchyma [61,62]. Soluble Aβ oligomers disrupt synaptic signalling, impair long term potentiation, and interfere with neuronal communication, contributing to early synaptic dysfunction before neuronal loss occurs [63]. Cerebral amyloid accumulation occurs years before the onset of clinical symptoms and is a defining character of preclinical AD. Amyloid burden can be assessed in vivo using PET imaging or cerebrospinal fluid biomarkers [64].

The Centiloid (CL) scale is used to quantify PET amyloid burden, enabling standardised comparison across tracers and imaging platforms. Within this scale, values below approximately 20–25 CL are considered amyloid negative [65,66], whereas higher Centiloid values indicate increasing cerebral amyloid deposition and greater likelihood of underlying AD pathology. This quantitative approach allows stratification of individuals along the preclinical AD continuum and facilitates comparison across cohorts and studies [67,68]. Emerging evidence suggests that peripheral metabolic and inflammatory signals may influence amyloid accumulation during early disease stages [69,70]. Alterations in gut microbial composition and microbial metabolites, including SCFAs, have been shown to modulate immune and inflammatory pathways that interact with amyloid-related processes [71]. Although causal relationships remain to be fully established, these observations provide a rationale for examining gut microbiota and microbial function in relation to cerebral amyloid burden, particularly within the preclinical phase of AD [69,72].

### 3.2. Tau Pathology

Tau is a microtubule associated protein responsible for stabilising axonal cytoskeletal structures [73]. In AD, tau undergoes hyperphosphorylation and conformational changes leading to misfolding and aggregation into intracellular neurofibrillary tangles [74]. These tangles impair neuronal transport systems, disrupt mitochondrial function, and contribute to synaptic degeneration and neuronal loss [75]. Tau pathology shows a strong association with cognitive impairment and clinical symptom severity, particularly at later disease stages, and is closely linked to neurodegeneration [76]. Evidence suggests that tau pathology may be present at low levels during preclinical stages, although its burden increases substantially with disease progression [77,78]. Recent studies indicate that inflammatory and metabolic processes influence tau phosphorylation and aggregation.

Neuroinflammatory signalling, including microglial activation, has been implicated in promoting tau pathology, suggesting potential interactions between central immune responses and peripheral biological factors, including gut-derived metabolites. SCFAs have been reported to influence tau phosphorylation indirectly through their effects on microglial activation and systemic inflammatory signalling, both of which are implicated in tau-related neurodegeneration [77,79].

### 3.3. Neuroinflammation

Neuroinflammation is a key pathological feature of AD and is characterised by sustained activation of microglia, reactive astrocytosis, and increased pro-inflammatory cytokine signalling within affected brain regions [76]. In the early stages of disease, microglia and astrocytes may play protective roles by facilitating clearance of Aβ and supporting neuronal homeostasis [80]. However, as pathology accumulates, glial cells can adopt chronically activated phenotypes that promote the release of cytokines, chemokines, and reactive oxygen and nitrogen species. This persistent inflammatory environment disrupts synaptic function, impairs neuronal communication, and contributes to progressive neuronal injury and loss [81,82]. Neuroinflammatory responses are closely linked to both amyloid and tau pathology. Aβ aggregates and hyperphosphorylated tau directly activate microglia and astrocytes through pattern recognition receptors, resulting in the amplification of inflammatory signalling cascades. Several risk genes, including triggering receptor expressed on myeloid cells 2 (TREM2) and cluster of differentiation 33 (CD33), are also implicated in microglial activation and inflammatory regulation [45]. Together, these findings suggest that neuroinflammation is not merely a secondary consequence of protein aggregation but an active contributor to disease progression that interacts with other pathological processes [83].

Importantly, neuroinflammation also represents a pathway through which peripheral biological processes may influence central nervous system pathology. Emerging evidence indicates that gut microbial composition and gut-derived metabolites, including SCFA, can modulate immunity and inflammatory signalling [29,84]. These peripheral immune signals may affect microglial activation states and neuroinflammatory responses, providing a potential mechanistic link between the gut environment and brain pathology in AD [85,86]. While the precise pathways remain to be fully elucidated, these observations highlight neuroinflammation as an integrative process bridging central and peripheral contributors to AD pathophysiology [86,87].

### 3.4. Oxidative Stress

Oxidative stress results from the excessive production of reactive oxygen species (ROS) combined with insufficient antioxidant defence systems [88]. ROS-mediated damage affects lipids, proteins, nucleic acids, and mitochondria, particularly in metabolically active neuronal regions [88]. Both amyloid and tau pathology are associated with increased oxidative damage, which contributes to neuroinflammatory activation and synaptic dysfunction [89]. Age-related mitochondrial impairment further increases neuronal vulnerability to oxidative injury, supporting a role for oxidative stress across multiple stages of AD progression [90,91]. Gut microbial composition and gut-derived metabolites, particularly SCFA, influence systemic oxidative and inflammatory pathways. SCFAs, particularly butyrate, have been reported to reduce oxidative damage by enhancing antioxidant enzyme activity and limiting excessive reactive oxygen species production in intestinal and systemic tissues, providing a plausible pathway linking gut microbial metabolism to oxidative processes relevant to AD [92].

## 4. Risk Factors, Diagnosis, and Therapeutic Context in Alzheimer’s Disease

AD is influenced by both non-modifiable and modifiable factors, including age, genetic susceptibility (particularly apolipoprotein E ε4 (APOE) ε4), metabolic health, and lifestyle behaviours such as diet and physical activity. Age is the strongest known risk factor for AD, and APOE ε4 is the most significant genetic risk factor, with carriers showing increased AD risk and earlier disease onset [28,93]. Increasing evidence suggests that several modifiable risk factors associated with AD risk are closely linked to gut microbial composition and function. Diet-related metabolic changes, chronic inflammation, and immune dysregulation, all of which are influenced by the gut microbiome, may contribute to early pathological processes during the preclinical stage of AD. Emerging evidence also points to an interaction between APOE genotype and gut microbial composition, suggesting that APOE ε4 carriers may respond differently to diet and microbiome-related interventions [94,95].

### 4.1. Diagnosis and Preclinical Alzheimer’s Disease

Recent advances have enabled AD to be identified using biological markers rather than relying only on clinical symptoms [96]. Amyloid imaging and cerebrospinal fluid measures allow the detection of disease-related pathology in cognitively unimpaired individuals [32,97]. This preclinical stage involves early amyloid accumulation with no neurodegeneration, consistent with the definition given above. Studying biological changes during this stage is important, as early alterations are less likely to reflect consequences of advanced disease and may identify changes in gut microbial composition and function. Standardised amyloid PET quantification, such as the Centiloid scale discussed above, allows individuals to be stratified along this preclinical continuum with precision, supporting more consistent identification of early biological change across cohorts [98].

### 4.2. Therapeutic Challenges and Early Disease Focus

Current treatments for AD provide limited clinical benefit once cognitive decline has been established. Commonly used symptomatic therapies, including the cholinesterase inhibitors donepezil, rivastigmine, and galantamine, as well as the N-methyl-D-aspartate (NMDA) receptor antagonist memantine, offer modest improvements in cognition and daily functioning but do not alter the underlying disease process [99,100,101]. More recently, disease modifying therapies such as the anti-amyloid monoclonal antibody lecanemab have been introduced for early-stage AD; however, their clinical benefits remain limited and are restricted to selected patient populations [102,103]. Most available therapies are therefore initiated only after substantial neuropathological damage has already occurred, reducing their capacity to meaningfully alter disease progression [104]. This therapeutic limitation highlights a major challenge in AD management and underscores the importance of more research and clinical attention toward earlier stages of the disease [105,106].

As a result, there is increasing interest in understanding the biological processes that occur during the preclinical phase of AD, when individuals remain cognitively unimpaired despite underlying pathology as defined above [1]. During this stage, inflammatory-, metabolic-, and immune-related processes are already active and may contribute to later neurodegeneration. These early biological changes represent potential targets for intervention before irreversible neuronal damage develops [107]. Emerging evidence suggests that peripheral systems, including the gut microbiome, may influence these early inflammatory and metabolic pathways through gut–brain communication mechanisms [78]. Alterations in gut microbial composition and microbial metabolites have been linked to immune modulation, metabolic regulation, and neuroinflammatory signalling, all of which are relevant to AD pathophysiology [13,108]. Understanding how these peripheral processes interact with early brain pathology may provide new opportunities for identifying individuals at risk and for developing preventative or disease-modifying strategies [109]. Focusing on early disease mechanisms therefore offers a promising pathway for improving AD outcomes. By targeting biological changes that occur prior to clinical symptom onset, future therapeutic approaches may support risk reduction, delay disease progression, and ultimately reduce the burden of AD [110,111].

### 4.3. Emerging Gut Microbiome Concepts in Alzheimer’s Disease

The gut microbiome is identified as an important regulator of immune, metabolic, and inflammatory processes relevant to brain health [112]. Through the formation of microbial metabolites, modulation of immune signalling, and interaction with host metabolic pathways, gut microorganisms contribute to systemic homeostasis and gut–brain communication [3,109]. Alterations in gut microbial composition and metabolic output have been reported in ageing populations and in several neurodegenerative disorders, suggesting that the gut microbiome may play a role in CNS [3].

In AD, growing evidence indicates that gut microbial dysbiosis is associated with neuroinflammatory activity, metabolic dysfunction, and amyloid-related processes [113,114]. However, much of the current literature focuses on individuals with established cognitive impairment or dementia. As a result, it remains unclear whether observed microbial alterations represent contributing factors to disease development or secondary consequences of advanced pathology, medication use, dietary changes, or functional decline [115,116]. Although evidence specific to preclinical AD remains limited, the gut microbiome is a highly modifiable biological system that may influence early disease-related processes [117]. Dietary intake, lifestyle factors, and pharmacological interventions can alter gut microbial composition and metabolic output, highlighting the potential for preventative or risk-modifying strategies [116,118].

Investigating the gut microbiome during the preclinical stage allows the assessment of microbial changes before the onset of cognitive symptoms, when neuropathological processes are already underway but clinical manifestations have not yet emerged [119]. Studying gut microbial characteristics at this early stage also reduces the likelihood that observed differences are confounded by advanced disease effects, such as medication exposure, altered mobility, or significant dietary changes [81,120]. Consequently, preclinical investigation provides a more reliable opportunity to identify gut microbiome-related alterations that may be linked to early AD pathology rather than to disease complications [121].

Together, these considerations highlight the importance of focusing on gut microbiome-related changes during the earliest stages of AD. Such an approach may improve the understanding of disease mechanisms, support early risk identification, and improve future strategies aimed at modifying disease trajectories before irreversible neurodegeneration occurs [122,123].

## 5. The Gut–Brain Axis

The gut–brain axis refers to the bidirectional communication between the gastrointestinal system and the central nervous system. This communication is mediated through neural, immune, endocrine, and microbial pathways and plays an important role in regulating physiological processes such as metabolism, immune activity, and brain function [124,125]. Within this network, the gut microbiome acts as an active biological regulator rather than a passive microbial community [126].

**Figure 2:**
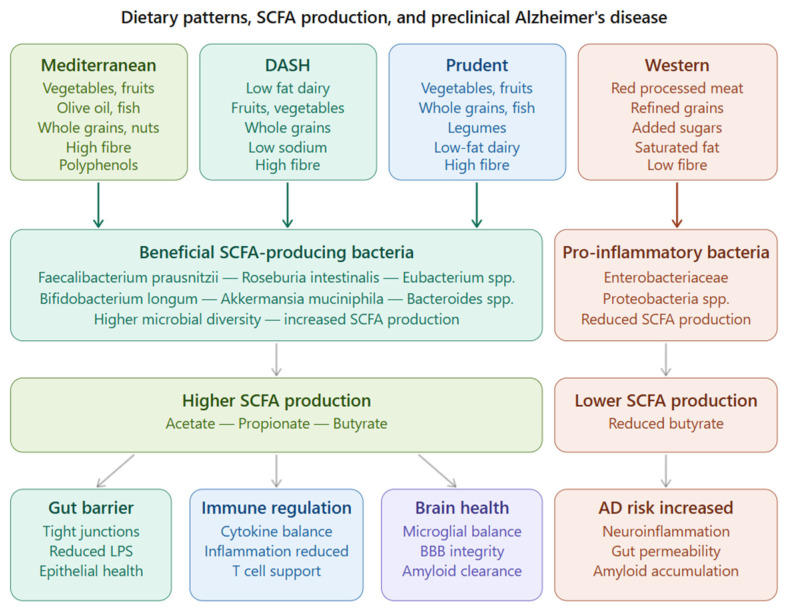
Overview of the gut–brain axis in preclinical Alzheimer’s disease. Bidirectional communication between the gut microbiota and the brain occurs through neural, immune, endocrine, and metabolic pathways. Gut-derived signals, including short-chain fatty acids, cytokines, and microbial metabolites, influence neuroinflammatory and metabolic processes relevant to early AD pathology. The lower panels illustrate dietary- and SCFA-related factors associated with gut–brain axis activity. Abbreviations: SCFAs, short-chain fatty acids; AD, Alzheimer’s disease; HPA, hypothalamic–pituitary–adrenal axis; BBB, blood–brain barrier.

Microbial-derived signals and metabolites influence immune cell activity, metabolic regulation, and neural signalling, thereby modifying peripheral environments that affect brain function [127]. Through these mechanisms, alterations in microbial composition can modify inflammatory and metabolic conditions in ways that may increase vulnerability to neurodegenerative processes [109,128,129]. These interactions position the gut microbiome as a potential contributor to biological pathways involved in AD (Figure 2).

The influence of the gut microbiome on brain function is conveyed through several interconnected communication routes. Neural signalling occurs primarily via the vague nerve, which provides a direct anatomical link between the gut and the brain and responds to microbial activity [130,131,132]. In parallel, immune pathways transmit microbial signals through the activation of peripheral immune cells and the release of cytokines and inflammatory mediators, which can influence neuroinflammatory activity within the central nervous system [133,134,135]. Endocrine signalling further integrates hormonal responses with neural and immune regulation, allowing systemic physiological changes to affect brain function [136].

Microbial metabolites are also another important component of gut–brain communication. SCFAs, produced through bacterial fermentation of dietary substrates, are among the most extensively studied of these metabolites [137]. SCFAs regulate epithelial barrier integrity, immune cell differentiation, cytokine production, and metabolic pathways. Through these actions, SCFAs can modulate neuroinflammatory processes and neuronal function, providing a direct biochemical link between gut microbial activity and brain health. The specific mechanisms through which SCFAs act on immune and neuroinflammatory pathways are discussed in detail in Section 6 [130]. Although the current findings are not yet conclusive, growing evidence suggests that the disruption of gut–brain communication may contribute to the inflammatory and metabolic disturbances observed during the early stages of AD [133,138]. Alterations in microbial composition and metabolite production may therefore influence disease-related pathways well before clinical symptoms become apparent. Clarifying the involvement of the gut–brain axis in these early processes is important for understanding the contribution of peripheral biology to AD pathophysiology and for identifying potential targets for early intervention [113,139].

### 5.1. Gut Microbial Changes in Neurodegenerative Diseases

Alterations in gut microbial composition and function have been reported across several neurodegenerative disorders, including Parkinson’s disease (PD) and AD [140,141], with PD included here as a comparative example rather than a primary focus of this review. Studies consistently describe changes in microbial taxa, microbial metabolic pathways, and differences in microbial metabolite production that are associated with inflammatory, immune, and metabolic processes [13,81,140]. These findings support the concept that gut microbial dysregulation is linked to neurodegenerative pathology.

In PD, gut microbial alterations have been observed even before the onset of motor symptoms, suggesting that microbial changes may occur early in disease development [142,143]. Similar patterns have been reported in AD, including the reduced abundance of beneficial SCFA-producing taxa, altered fermentation-related pathways, and differences in faecal metabolite profiles [5,6]. However, most AD-related studies have focused on individuals with mild cognitive impairment or established dementia, and studies examining gut microbial alterations specifically during the preclinical stage of AD remain scarce [79,144].

Available findings are also not yet consistent across studies. Variability in study design, sequencing methods, dietary assessment, and participant characteristics further complicates interpretation [145]. As a result, it remains unclear whether observed microbial differences represent early disease-related changes or secondary effects of cognitive decline, medication use, or lifestyle alterations.

These limitations highlight the need for well-designed studies that focus on cognitively unimpaired individuals stratified by underlying AD pathology. Investigating gut microbial composition, functional pathways, and metabolite production during the preclinical stage may provide critical insight into early biological changes associated with AD and help clarify the potential role of the gut microbiome in disease initiation and progression [121,145,146].

### 5.2. Evidence for Microbiome Dysregulation in Alzheimer’s Disease

Accumulating evidence indicates that AD is associated with significant alterations in gut microbial composition and function [147]. These microbial changes are characterised by a reduction in commensal taxa involved in metabolic homeostasis and an abundance of taxa linked to pro-inflammatory activity. Together, these changes suggest the disruption of key microbial functions related to fermentation, immune regulation, and host metabolic balance in AD [82,147,148]. One of the most consistently reported findings in AD and cognitive impairment is a reduced abundance of SCFA-producing genera, including *Faecalibacterium*, *Roseburia*, *Eubacterium*, and *Butyricicoccus* [148]. These taxa are major contributors to butyrate and propionate production and play important roles in maintaining intestinal barrier integrity, regulating immune responses, and supporting metabolic homeostasis. Their depletion has been linked to increased gut permeability, enhanced systemic inflammation, and metabolic dysregulation [149,150].

In contrast, several studies report an increased relative abundance of taxa such as Escherichia/Shigella and other members of the Proteobacteria phylum, which are commonly associated with inflammatory signalling, oxidative stress, and metabolic imbalance in ageing and AD populations (Table 1) [81,151]. The increased abundance of these taxa further supports the presence of a pro-inflammatory microbial environment in individuals with AD pathology [152].

Beyond taxonomic changes, functional alterations in microbial metabolism have also been reported. These include disruptions in carbohydrate fermentation pathways, reduced SCFA biosynthesis, and changes in amino acid and lipid metabolism [71]. SCFAs are critical mediators of immune modulation, intestinal barrier function, and systemic metabolic signalling [153]. Consequently, reduced SCFA availability has been associated with reduced inflammatory responses and impaired metabolic regulation, processes that are closely linked to AD pathophysiology [6].

Importantly, most evidence has been derived from symptomatic populations; a small number of studies suggest that gut microbial alterations may also be detectable during the preclinical stage of AD [154,155]. Early changes in SCFA-producing taxa and microbial metabolic pathways have been reported in cognitively unimpaired individuals with biomarker evidence of cerebral amyloid pathology [151,156]. While these findings are not yet consistent across studies, they indicate that microbial dysregulation may begin during the earliest phases of AD pathology.

Overall, the current evidence indicates that gut microbial dysbiosis in AD involves both taxonomic and functional alterations, although their relevance during the preclinical stage remains unclear.

**Table 1:** Key gut microbial taxa and their functional roles relevant to neurodegenerative processes.

Microbial Group/Taxa	Primary Functional Role	Major Metabolites Produced	Potential Relevance to AD
***Faecalibacterium*** **spp.**	SCFA fermentation of dietary fibre	Butyrate	Anti-inflammatory, supports gut barrier integrity, often reduced in AD [156,157]
***Roseburia*** **spp.**	SCFA-producing commensals	Butyrate	Associated with neuroprotective functions and reduced in cognitive decline [158]
***Bacteroides*** **spp.**	Carbohydrate metabolism and acetate generation	Acetate	Altered in early cognitive impairment and linked with metabolic imbalance [157,159]
***Akkermansia*** **spp.**	Mucin degradation and epithelial maintenance	Acetate, propionate	Reduced abundance associated with metabolic ageing and immune imbalance [159,160]
***Lachnoclostridium*** **spp.**	Proteolytic fermentation	Inflammatory metabolites	Increased in AD populations and linked with pro-inflammatory states [152,161]
***Enterobacteriaceae family***	Endotoxin production	LPS	Elevates systemic inflammation and may accelerate neuroinflammatory processes [162,163]

## 6. Microbial By-Products, Their Biological Functions, and Relevance to Alzheimer’s Disease

In addition to alterations in microbial composition, the metabolic output of the gut microbiome plays a critical role in host physiology. Microbial metabolites generated through fermentation act as key signalling molecules linking gut microbial activity with immune regulation, metabolic control, and neural function [72,164]. These metabolites provide direct evidence of microbial metabolic activity that cannot be inferred from taxonomic data alone [72].

The investigation of microbial metabolic products therefore offers important insight into how gut microbial activity may influence biological processes associated with disease development, particularly during early pathological stages [6,153].

### 6.1. Microbial Fermentation and Generation of Metabolic By-Products

Gut microbial fermentation is the primary process through which microbial by-products are generated in the large intestine. Gut microbiota ferment undigested dietary fibres, resistant starches, and oligosaccharides in the large intestine, generating metabolic by-products that include SCFAs, gases, and other organic acids [4,165]. The type and quantity of these metabolites depend on substrate availability, microbial composition, and metabolic interactions within the microbial community [166]. Distinct bacterial taxa contribute to the production of specific fermentation products. Members of the Firmicutes phylum, including *Faecalibacterium prausnitzii* and *Roseburia* species, are primary butyrate producers, while *Bacteroides* species within the Bacteroidetes phylum predominantly generate acetate and propionate through carbohydrate fermentation pathways [6,167]. Metabolic cross-feeding between microbial groups further influences fermentation outcomes, as acetate produced by one group may serve as a substrate for butyrate-producing bacteria, supporting microbial ecosystem stability and energy utilisation [157].

In addition to energy extraction, microbial fermentation produces metabolites that function as signalling molecules influencing immune, metabolic, and neural pathways involved in ageing and neurodegenerative disease [168]. Accordingly, alterations in microbial fermentation or substrate availability can modify the profile of microbial by-products, with potential consequences for systemic inflammation and brain-related biological processes [154,169].

### 6.2. Short-Chain Fatty Acids as Functional Mediators of Host Biology

SCFAs are microbial metabolites produced through the anaerobic fermentation of non-digestible carbohydrates in the colon and represent a major interface between gut microbial activity and host physiology [170,171]. Acetate, propionate, and butyrate are the predominant SCFAs, while branched SCFAs such as isobutyrate and valerate are present at lower concentrations [6,171]. After production, SCFAs exert both local effects within the intestinal environment and systemic effects following absorption into the circulation.

SCFAs play an important role in maintaining intestinal barrier integrity by supporting epithelial energy metabolism and function, thereby limiting the translocation of microbial products and inflammatory mediators [6]. In parallel, SCFAs regulate host immune responses through multiple mechanisms, including activation of G protein-coupled receptors such as GPR41 and GPR43 and modulation of histone deacetylase activity, leading to changes in gene transcription relevant to inflammatory and metabolic pathways [170,172]. Through these actions, SCFAs influence cytokine production, immune cell differentiation, and systemic inflammation. In addition to peripheral effects, SCFAs also participate in gut–brain communication [173]. Experimental studies demonstrate that a proportion of circulating SCFAs can access the central nervous system and influence microglial activity, neuronal energy metabolism, and neurotrophic signalling [174]. These findings support a role for SCFAs as important mediators linking gut microbial metabolism with immune regulation and neural function. Although acetate, propionate, and butyrate share overlapping functions, their effects are not identical; butyrate is the primary energy source for colonocytes and has the strongest evidence for anti-inflammatory action, while propionate is more closely linked to hepatic and metabolic signalling, and acetate serves mainly as a substrate for other microbial groups [6,171].

Despite this biological relevance, evidence directly linking SCFAs to AD pathology in humans remains limited, particularly during the preclinical stage [174,175]. Most current data are derived from experimental models or cross-sectional clinical studies. Further investigation is therefore, required to clarify how SCFA-mediated mechanisms contribute to early disease-related biological changes prior to the onset of cognitive impairment [153,176,177]. It is also increasingly recognised that SCFA effects are concentration- and context-dependent; while physiological concentrations generally support anti-inflammatory and barrier protective functions, excessive SCFA accumulation has been associated with adverse immune and inflammatory responses in some experimental models, highlighting the need for a balanced interpretation of SCFA-related findings [174,175].

### 6.3. Immune, Metabolic, and Neuroinflammatory Effects of SCFAs

SCFAs play an important role in regulating immune and metabolic processes relevant to brain health [2]. SCFAs influence immune cell activity by modulating cytokine production and promoting immune tolerance, particularly through effects on regulatory T cells and multiple innate and adaptive immune cells (including dendritic cells, macrophages, neutrophils, B cells, and CD4^+^ and CD8^+^ T cells) [178]. These actions contribute to the maintenance of systemic immune balance and help limit excessive or chronic inflammatory responses.

In addition to their immune effects, SCFAs are important regulators of host metabolism [2]. Acetate, propionate, and butyrate act as signalling molecules that influence glucose metabolism, lipid homeostasis, and overall energy balance. By interacting with host metabolic pathways, SCFAs link gut microbial activity with peripheral metabolic regulation [179]. Disruption of these pathways has been associated with metabolic dysfunction, insulin resistance and increased inflammatory activity [5,176], all of which are recognised as risk factors for neurodegenerative diseases.

SCFAs also play a role in regulating neuroinflammatory processes through both indirect and direct mechanisms. Circulating SCFAs affect blood–brain barrier integrity and modulate microglial activation, thereby influencing inflammatory responses within the central nervous system [180]. A reduced availability of SCFAs has been linked to increased neuroinflammatory signalling and impaired neuronal support functions [181]. Together, these findings indicate that SCFAs act as key mediators connecting gut microbial metabolism with immune regulation, metabolic control, and neuroinflammatory processes relevant to AD [5]. However, evidence describing SCFA-mediated effects specifically during the early preclinical stages of AD remains limited. Most available data are derived from animal models or cross-sectional human studies [121,182]. Further investigation in preclinical populations is therefore required to clarify how SCFAs contribute to early immune, metabolic, and inflammatory alterations associated with AD pathology.

### 6.4. Alterations in Microbial By-Products in Ageing and Alzheimer’s Disease

Ageing is associated with alterations in gut microbial composition and metabolic output, including reductions in SCFA production [137]. Similar changes have been reported in mild cognitive impairment and AD, where a decreased abundance of SCFA-producing genera such as *Faecalibacterium* and *Roseburia* coincides with altered microbial fermentation pathways and reduced availability of butyrate and propionate [156]. These metabolic alterations are linked to inflammatory and metabolic dysregulation relevant to AD pathophysiology [183]. However, evidence specifically addressing alterations in microbial by-products during the preclinical stage of AD remains limited. Only a small number of studies have reported changes in SCFA-producing taxa and microbial metabolic pathways in cognitively unimpaired individuals with biomarker evidence of amyloid pathology [177,184]. These findings suggest that microbial metabolic dysregulation may begin prior to the onset of clinical symptoms, although further investigation is required to confirm the timing and significance of these early changes.

### 6.5. Diet as a Modifiable Determinant of Gut Microbiota and SCFA Production in Alzheimer’s Disease

Diet is associated with cognitive health and AD risk, with many studies showing that healthier dietary patterns are linked to slower cognitive decline and lower rates of AD. Diet influences inflammation, metabolic health, and blood vessel function, which are all important in the development of AD [185]. Mediterranean, DASH, and prudent dietary patterns have been associated with improved cognitive performance and reduced brain atrophy, whereas Western dietary patterns have been linked to poorer cognitive outcomes and increased AD risk [186,187,188]. Diet also plays a main role in determining gut microbial composition, diversity, and metabolic function. Diets rich in dietary fibre are associated with an increased abundance of SCFA-producing genera such as *Faecalibacterium*, *Roseburia*, and *Eubacterium*, whereas diets high in saturated fat and refined carbohydrates are linked to reduced microbial diversity and enrichment of pro-inflammatory taxa [189,190]. Polyphenol-rich foods further promote the growth of beneficial genera, including *Akkermansia* and *Bifidobacterium*, which are associated with improved metabolic and immune regulation [188,191]. Studies have also shown that high fibre- and plant-based diets are associated with higher faecal concentrations of butyrate and propionate, while Western dietary patterns are linked to reduced SCFA production. Dietary intervention studies demonstrate that SCFA profiles change within weeks of dietary modification, highlighting the rapid responsiveness of gut microbial metabolism to dietary intake [192].

These diet-dependent microbial and metabolic changes are biologically relevant to AD. SCFAs influence intestinal barrier integrity, systemic inflammation, insulin sensitivity, and neuroinflammatory signalling, all of which are implicated in AD pathophysiology [71]. Epidemiological studies further report that dietary patterns associated with higher SCFA production are also linked to reduced systemic inflammation and improved metabolic health, suggesting a mechanistic pathway connecting diet, gut microbiota, SCFAs, and brain health [193]. Despite this growing evidence, relatively few studies have examined dietary patterns alongside gut microbial composition, microbial functional pathways, and SCFA profiles within the same cognitively unimpaired, amyloid-stratified population. This gap limits the understanding of how diet-related microbial and metabolic changes relate to early AD pathology [4,7].

## 7. Dietary Patterns Associated with Gut Microbiota and SCFA Profiles

Dietary patterns are key modulators of gut microbial composition and SCFA production, thereby influencing microbial metabolic activity and host metabolic health. These effects relate to the mechanisms outlined in Section 6.5, involving substrate availability for microbial fermentation and SCFA production.

### 7.1. Mediterranean and DASH Dietary Patterns

The Mediterranean (MeDi) and Dietary Approaches to Stop Hypertension (DASH) diets are two well-researched dietary models associated with improved metabolic, cardiovascular, and cognitive outcomes in ageing populations. Both dietary models emphasise the high consumption of plant-based foods, including vegetables, fruits, legumes, whole grains, and nuts, while limiting red and processed meats, saturated fats, and added sugar [194,195,196]. The Mediterranean dietary pattern is further characterised by the use of extra virgin olive oil as the primary source of fat, moderate intake of fish and poultry, and low to moderate consumption of dairy product [197].

Both dietary patterns influence gut microbial ecology and metabolic output, primarily through the availability of fermentable fibres, polyphenols, and unsaturated fatty acids [198]. These components promote the growth of beneficial commensals such as *Faecalibacterium prausnitzii*, *Roseburia intestinalis*, and *Bifidobacterium longum*, which are major producers of butyrate and other SCFAs [113,159,199].

In addition to their metabolic benefits, adherence to the Mediterranean and DASH dietary patterns has been associated with lower systemic inflammation and reduced oxidative stress, both of which are closely linked to AD pathophysiology [200]. Higher adherence to these diets has also been related to reduced cerebral amyloid burden, improved vascular function, and slower rates of cognitive decline in older adults [201]. The MIND diet, which combines elements of the Mediterranean and DASH patterns, has similarly been associated with the slower accumulation of AD-related pathology and reduced markers of cerebral small vessel disease [200,202].

Increasing evidence also suggests that the gut microbiome may partly mediate these protective effects. Diet-driven changes in microbial composition and SCFA production can influence neuroinflammatory signalling, vascular health, and neuronal energy metabolism, providing a potential biological pathway linking dietary patterns with brain health and AD risk [202]. It should be noted that few studies have directly assessed diet, gut microbiota, and amyloid pathology together within the same preclinical population; much of the current evidence linking these factors is therefore inferential rather than directly demonstrated [71,193].

### 7.2. Prudent Dietary Pattern

The prudent dietary pattern is characterised by a higher intake of vegetables, fruits, whole grains, fish, legumes, and low-fat dairy. This pattern has been associated with improved metabolic and inflammatory profiles in ageing populations. Prudent diets support higher microbial diversity and enrichment of SCFA-producing species [203,204].

### 7.3. Western Dietary Pattern

In contrast, the Western dietary pattern is characterised by a high intake of red and processed meats, refined grains, added sugars, saturated fats, and ultra-processed foods. This is linked to reduced microbial diversity, lower abundance of beneficial commensals, and lower SCFA production [205,206]. Western diets also promote the pro-inflammatory taxa and increased gut permeability, contributing to metabolic dysregulation and systemic inflammation [12,207]. This results in immune activation, oxidative stress, and altered energy metabolism [208].

## 8. Conclusions

The current evidence suggests that changes in gut microbial composition and metabolic activity are associated with early Alzheimer’s disease pathology. A reduced abundance of SCFA-producing taxa, altered microbial fermentation pathways, and disruption of gut–brain communication have been reported across ageing and neurodegenerative disease, and similar patterns are beginning to emerge in cognitively unimpaired individuals with biomarker evidence of amyloid pathology. Diet, particularly through its effect on microbial substrate availability and SCFA production, appears to be an important modifiable factor linking these processes, with Mediterranean, DASH, prudent, and MIND dietary patterns generally associated with beneficial microbial profiles, and Western dietary patterns associated with reduced microbial diversity and increased inflammatory activity.

This evidence base has important limitations. Most studies have been conducted in symptomatic AD or MCI populations rather than cognitively unimpaired biomarker-confirmed individuals, and much of the available evidence is cross-sectional, limiting conclusions about the direction of the relationship between microbial change and disease progression. Sequencing approaches have also generally been limited to genus level, providing limited insight into the specific microbial species and functional pathways involved. Diet, gut microbiota, and microbial metabolites are rarely investigated together within the same study population, and few studies have directly assessed diet, gut microbiota, and amyloid pathology together in preclinical cohorts. As a narrative review, this work is also subject to its own limitations, including a focus on SCFAs as the primary microbial metabolite class; other potentially relevant pathways, including intestinal barrier markers such as zonulin and LPS, TMAO, bile acids, tryptophan metabolites, and microbially derived neurotransmitters, were not covered in depth.

Future research should prioritise longitudinal species-level metagenomic studies in amyloid-stratified cognitively unimpaired populations that combine microbial functional pathway profiling, SCFA measurement, and dietary assessment within the same cohort. Further investigation is also needed into faecal microbiota transplantation as a potential intervention in AD models, the relationship between intestinal barrier function and amyloid pathology, and the contribution of additional dietary patterns such as ketogenic and fasting-based approaches. Resolving these questions is central to determining whether the gut microbiome can serve as a meaningful early indicator of Alzheimer’s disease risk, and whether microbiome or diet-based strategies could meaningfully contribute to early intervention before irreversible neurodegeneration occurs.

## Data Availability

No new data were created or analyzed in this study. Data sharing is not applicable to this article.
